# From Nano- to Microsilver: Morphology Control and Shape Evolution of Facile One-Step Electrochemical Synthesis of Silver Particles on TiO_2_ Nanotubes

**DOI:** 10.1021/acs.langmuir.5c01022

**Published:** 2025-06-20

**Authors:** Marta Nycz, Katarzyna Arkusz

**Affiliations:** Department of Biomedical Engineering, Faculty of Engineering and Technical Sciences, 49792 University of Zielona Gora, Prof. Z. Szafrana 4, Zielona Gora 65-516, Poland

## Abstract

Nano- and microsilver (Ag) exhibit a wide range of desirable catalytic, electronic, adsorption, and antimicrobial features, complementing the excellent properties of titanium dioxide (TiO_2_) nanotubes by increasing their specific surface area. Formed via electrodeposition processes, it has advantages such as high purity; the methodology is repeatable, reproducible, fast, and easy. Anodically produced TiO_2_ nanotubes were modified with nano- and microsilver by electrodeposition from silver nitrate solution using cyclic voltammetry and chronoamperometry. The mechanisms of electrodeposition and evolution of silver morphology were analyzed based on cyclic voltammograms/current density–time curves and micrographs taken using a scanning electron microscope. The variables were the AgNO_3_ precursor solution’s concentration, applied voltage, and number of cycles or deposition time. It was proven that there is a universal mechanism for the evolution of the morphology of the deposited silver, regardless of the electrodeposition method used. With the increase of the precursor concentration, deposition time, and applied voltage, the silver grows and reorients according to the following scheme: initially deposited Ag has the form of clusters of Ag nanoparticles resembling nanoflowers, nanoleaves, and spherical nanoparticles, which over time through aggregation create short dendrites and then develop subsequent multilevel branches to evolve into interconnected microsized particles.

## Graphical abstract

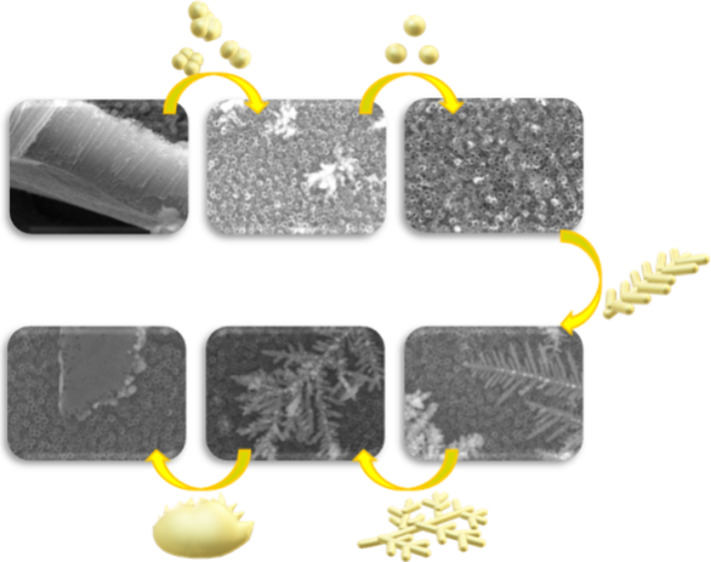

## Introduction

Vertically oriented TiO_2_ nanotubes (TNTs) prepared on a metal substrate by electrochemical anodization since they were discovered in 2001 ^1^ have enjoyed unwavering interest, especially in biomedical applications. Currently, the aim of nanotechnology is to combine nanomaterials in order to increase their benefits.^2^ TiO_2_ nanocomposite structures doped with noble metals, such as ruthenium,^3^ gold,^4^ silver,^4^ and platinum^4^ or palladium,^5^ have attracted great interest in this context. They are used in electrochemical sensors due to their increased specific surface area,^4^ excellent conductivity,^4^ and extraordinary electrocatalytic properties.^6^ Among these materials, silver is used the least frequently.^7–9^ However, the conductivity of silver is the best, and the price is much lower than that of the other noble metals mentioned above.^4,9,10^ In addition, Ag is often used to modify TiO_2_ materials in catalysis and photocatalysis, lithium-ion batteries, dye-sensitized solar cells, and various applications in implantology due to its biocidal properties.^9,11^ The properties of dopants can be shaped/improved for a specific application by controlling their size or shape.^12–14^ At the current stage of development, Ag nanocrystals can be synthesized across a broad size spectrum and with diverse morphologies, including cubes, octahedra, nanorods or nanowires, prisms, or plates, as well as icosahedral nanocrystals.^15^

Many methods for preparing nano- and microsilver-modified TNTs have been described, such as microwave-assisted approach,^16^ chemical^17^ and physical vapor deposition,^18^ photodeposition,^19^ electrodeposition,^20^ and chemical^11^ and biological^21^ reduction methods. In most cases, chemical reduction and photoreduction methods are used. However, these techniques involve toxic additives,^22^ and the process is difficult to control, which invariably leads to the disordered aggregation of particles on nanotubes.^10^ This affects the poor stability and process reproducibility of the resulting materials.^4,9^

It was also observed that photoreduced Ag cannot be strongly dispersed on the surface of TiO_2_ nanotubes.^23^ Therefore, the number of active sites on the Ag/TNT surface cannot significantly increase. Other methods, including sol–gel and ″wet″ chemical methods, where drying and heating are essential steps of the preparation process, are too complicated for large-scale production.^11^ On the other hand, electrochemical methods are cheap, highly productive, and repeatable.^24^ Deposition by reduction of metal ions from the electrolyte can be performed in two ways: either by supplying electrons from an external power source or through a currentless process, in which deposition occurs due to a reducing agent in the electrolyte. The former offers easier control and requires only a silver precursor, eliminating the need for auxiliary substances. This contributes to the high purity of the structures produced. The literature on the synthesis of Ag on TNTs by electrodeposition methods remains limited.^7,9,25–29^

In most of these reports, an aqueous solution of silver nitrate (AgNO_3_) salt is used as a silver precursor. The popularity of using AgNO_3_ is attributed to its low price^30^ and chemical stability^30^ compared to other silver salts. The precursor solution concentration reported in the literature ranges from 0.75 mM^27^ through 1 mM,^7^ and 10 mM,^26,28^ and 50 mM.^25^ The additives used include KNO_3_,^25^ NaNO_3_,^26^ thiopropionic acid,^31^ NaClO_4_,^31,32^ ethylenediamine,^31,32^ ethylene glycol,^33^ and HNO_3_,^34^ which can increase the efficiency of the process but also contaminate the produced particles. Among electrochemical deposition methods, galvanostatic electrodeposition, potentiostatic electrodeposition, pulse voltammetry, and cyclic voltammetry methods are commonly used. Potentiostatic electrodeposition can be divided into cathodization in the potential range from −0.4 V^9^ through −0.7 V^25^ and −1.0 V^28^ to −1.1 V^35^ and anodization at a potential of 0.8 V,^36^ and in the range of 30 to 50 V.^37^ Galvanostatic electrodeposition was used at current densities of 0.2 ^38^ and 0.3 ^27^ mA·cm^–2^. The cyclic voltammetric reduction was performed from −1.25 to −0.7 V.^18,20,39^

However, despite growing research interest, the mechanism of silver growth on TNTs, including the analysis of morphological changes with changes in basic process parameters, has not been performed so far. Some studies have analyzed only the influence of relatively narrow ranges of single process variables. For example, Baran and Yazici^27^ investigated the effect of time on the effects of deposition at 0.3 mA·cm^–2^ in 0.75 mM AgNO_3_ in the range of 240 to 1800 s. Similarly, Sang et al.^40^ carried out Ag deposition on TNTs from a solution of 0.01 M AgNO_3_ and 0.1 M NaNO_3_ at −15 mA·cm^–2^ of pulse current at the various values of 300, 600, and 1200 mC·cm^–2^.

This study aimed to elucidate the mechanisms governing the morphological evolution of electrodeposited silver by employing scanning electron microscopy (SEM), thereby establishing a correlation between the morphological characteristics and electrochemical behavior. The influence of silver nitrate concentration, deposition time, and applied voltage during chronoamperometric (CA) deposition of silver on the morphology of the resulting silver–titanium nanotube (Ag/TNTs) composites was systematically investigated. Subsequently, optimal conditions for cyclic voltammetric (CV) deposition of silver were determined, focusing on the effects of the silver precursor concentration and the number of deposition cycles on the morphology of the obtained Ag/TNT composites. This comprehensive analysis facilitated the proposal of a universal mechanism for the morphological evolution of electrochemically deposited silver, enabling the selection of experimental parameters conducive to the fabrication of composites with tailored structural properties.

## Materials and Methods

Titanium foil (99.7%), platinum mesh (99.9%), ammonium fluoride (NH_4_F, ≥98.0%), ethylene glycol (99.8%), and phosphate-buffered saline (PBS, 0.01 M, pH 7.4) were acquired from Sigma-Aldrich (St. Louis, Missouri, USA). Silver nitrate (99.9%) was supplied by Stanlab (Lublin, PL).

### Fabrication of TiO_2_ Nanotubes

Titanium dioxide (TiO_2_) nanotubes were formed on a titanium foil using the two-electrode system with a platinum mesh as a counter electrode in the electrochemical anodization process. Anodizing was carried out at 17 V for 62.5 min using an Autolab PGSTAT302N (Metrohm, Herisau, Switzerland) in ethylene glycol (85 wt %) with ammonium fluoride (0.65% wt.). The as-formed TNTs were heat-treated in an argon atmosphere at 450 °C for 2 h with heating and cooling rates of 6 °C·min^–1^ by using an AMP furnace (Zielona Gora, Poland).

### Electrodeposition of Silver on TiO_2_ Nanotubes

The deposition of silver was carried out using an electrochemical deposition process: chronoamperometry and cyclic voltammetry in a three-electrode system (Autolab PGSTAT302N, Metrohm, Herisau, Switzerland). The auxiliary electrode was a platinum mesh, and the reference electrode was a silver/silver chloride electrode (*E*_Ag/AgCl (3 M KCl)_ = 0.222 V vs SHE at 25 °C) by Metrohm. In the chronoamperometry method, the variable parameters were the AgNO_3_ precursor concentration, ranging from 0.1 to 50 mM (with conductivity ranging from 98.7 μS·cm^–1^ to 9.09 mS·cm^–1^), the deposition time, from 30 to 1200 s, and the voltage from −0.4 to −1.2 V. In the cyclic voltammetry method, the variable parameters were the AgNO_3_ precursor concentration ranging from 0.1 to 50 mM, and the number of deposition cycles from 5 to 75, at a constant potential range from −1.25 to −0.7 V with a scan rate of 50 mV·s^–1^. Then, the nanocomposite samples were rinsed with distilled water and dried under a nitrogen stream.

The paper presents current density–time transients for the chronoamperometric deposition process, current–potential transients for cyclic voltammetry, and charts of the dependence of the tested parameter (AgNO_3_ concentration, voltage, time, number of cycles) on the silver content in the obtained composites as well as the average value of the recorded current after its relative stabilization.

### Chemical and Morphological Characterization

The surface morphology of the samples was observed by using a scanning electron microscope (SEM, JEOL JSM-7600F, Tokyo, Japan). The elemental composition was examined by using an energy-dispersive X-ray spectroscopy detector (EDS, INCA, Oxford Instruments, Oxford, UK).

The silver content in Figures 3b, 5b, 7b, 9b, and 11b was presented as the mean and standard deviation of three randomly selected areas subjected to EDS analysis.

## Results and Discussion

SEM images (Figure 1) show the surface morphology of the TNTs after annealing. The heat treatment does not change the shape and dimensions of the nanotubes compared to the pure TiO_2_ nanotubes, as confirmed in previous studies.^18,20^ The nanotubes with dimensions of 1000 ± 68 nm in length and 50 ± 7 nm in outer diameter had a regular, cylindrical shape, were adjacent to each other by their edges, and had rings, the so-called bridges between neighboring nanotubes (Figure 1b), which result from the addition of water to the electrolyte used for anodizing. The bridges make the structure much more mechanically stable.^41^ The advantage of nanotubes produced in aqueous electrolytes is the high adhesion of the oxide to the metal substrate, which results in enhanced electron transport in applications where TNTs are used as an electrode material.^42^

**Figure 1:**
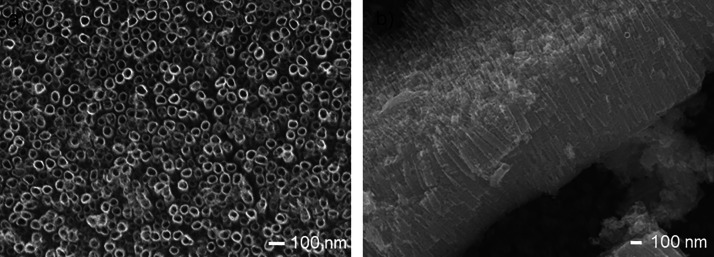
SEM images: (a) top view and (b) side view of TiO_2_-annealed nanotubes.

### The Influence of Precursor Concentration on the Morphology of Nanocomposite Produced by Chronoamperometry

SEM images (Figure 2) show the surface morphology of the Ag/TNT composite in which silver was deposited using the chronoamperometric method. The deposition process was conducted at a constant duration of 180 s and an applied voltage of −1.2 V, with variable concentrations of the silver ion precursor, silver nitrate, ranging from 0.1 to 50 mM. The addition of Ag (like annealing) does not cause any changes in the morphology and dimensions of the nanotubular structure, which was confirmed in previous studies.^7^ Silver deposited in the electrodeposition process in the form of spherical nanoparticles (Figure 2a,b) is located mainly on the surface of the nanotubes, on their edges, which results from the higher electric current density in these places while they are less frequently observed in the holes of TiO_2_ nanotubes and between them.^43^ The highly ordered structure of nanotubes is conducive to spontaneous self-assembly, i.e., uniform distribution of silver nanoparticles (AgNPs).

**Figure 2:**
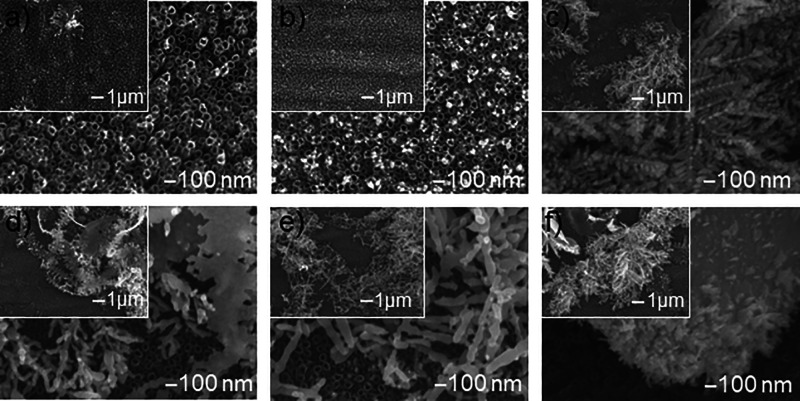
SEM images of the surface morphology of TNT nanocomposite samples with silver additive deposited by the chronoamperometric method at a constant deposition time of 180 s and a voltage of −1.2 V and variable concentrations of silver ion precursor AgNO_3_: (a) 0.1 mM, (b) 1 mM, (c) 5 mM, (d) 10 mM, (e) 25 mM, and (f) 50 mM.

SEM images (Figure 2c–f) show the evolution of the morphology of the deposited silver particles with the increase of the precursor concentration in the range of 5 to 50 mM. The increasing size of the deposited silver particles causes the clogging of the TNT channels, resulting in an obvious decrease in their specific surface area. Figure 2c shows the structure that contains interconnected dendritic particles. With the increase of the AgNO_3_ concentration, the dimensions of the formed dendrites increase through their growth laterally and vertically, and thus, they occupy the TNT surface area. Figure 2f shows the platform obtained from electrodeposition in a 50 mM solution that contained micrometric particles resembling the shape of a semicircle. These results are in agreement with the studies of Cheng et al.^44^ on the electrodeposition of nanosilver on a smooth ITO glass surface under a constant current density of 1 mA·cm^–2^ from a solution containing silver nitrate with concentrations ranging from 0.5 to 4 and 40 g·L^–1^ citric acid, in which the morphological evolution from initially formed rods with leaf-like branches gave rise to fractal structures with highly branched multilevel dendrites up to microhemispheres. Fractal growth is not an equilibrium, and the growth mechanism is explained by a diffusion-limited aggregation model. In these studies, it was decided to evaluate the effect of AgNO_3_ concentration and deposition time in a narrow range, i.e., 0.5–4 g·L^–1^ and 20–240 s, respectively, which is understandable due to the addition of citric acid, which can act as an additional reducing agent (apart from the reducing effect of electric current) but also as a functional capping agent and the selective adhesion to a particular plane of AgNPs.^45^ Yang et al.,^46^ in their studies on the effect of increased mass transport rate on the morphology of Au crystals electrochemically deposited on TNT, proved that higher conductivity of the precursor solution accelerates nucleation and particle growth rate, facilitating the formation of large-sized particles, including dendrites, which is also confirmed by the present studies. Moreover, the SEM images suggest that obtaining a uniform, layered coating of TNTs with silver using the CA method is impossible because the morphology of the TiO_2_ nanotubes determines this inhomogeneity.

The influence of the crystal structure on silver deposition is essential to note: studies by Liang et al.^11^ indicate that the anatase phase plays an important role in improving the bond strength between Ag and TiO_2_. Referring to own previous studies,^7,20^ it was proven that the TiO_2_ nanotube substrate annealed at 450 °C has a predominance of the anatase phase. Hence, it should be noted that the effects of silver deposition on TNTs with different crystal structures may differ both quantitatively and morphologically.

The formation of AgNPs and their deposition on TNTs involve the formation of silver nuclei at the surface of nanotubes due to the reduction of Ag^+^ ion by an applied potential. The concentration gradient of silver ions created between the solution and the TNT surface is the driving force for the movement of ions toward the electrode, as a result of which more free Ag^0^ atoms are formed on its surface, which collide with each other to form nuclei that become sites of nucleation and growth of nanoparticles as a result of further reduction of cations.^47^ The difference in charges of particles of different sizes can explain the agglomeration. Larger nanoparticles accept an electron from a neighboring smaller particle through a conductive substrate agglomerate, while smaller ones dissolve, a type of Ostwald ripening.^48,49^

The mechanism of metal nucleation and growth during electrodeposition is usually studied by chronoamperometry. The current density–time transients are shown in [Fig fig3]a. The initial seconds of the process are related to charging of the double layer. The initial rapid decrease in current density corresponds to the immediate nucleation process of the first Ag nuclei on the electrode and the growth of silver crystallites.^50^ In the case of lower concentrations of AgNO_3_ in the range of 0.1 to 5 mM, the current density stabilized after about 30 s, while for higher concentrations, the current density increased followed by its relative stabilization. The shape of the current density–time curves represents a typical nucleation process of 3D growth.^51^ The current density–time transients are analyzed using the Scharifker and Hills mathematical model (SH model).^52,53^ The model is commonly used to analyze the mechanisms of nucleation and growth and is applicable under the assumption that 3D growth of nuclei occurs and that the process is diffusion-controlled over an extended period. Theoretical current density–time transients predicted by the Scharifker–Hills models for the cases of instantaneous (dashed) and progressive (dotted) nucleation and growth are shown in inset Figure 3a. Instantaneous nucleation is characterized by the immediate formation and subsequent slow growth of all nuclei (all activated simultaneously). During progressive nucleation, nuclei are formed continuously with continually formed active sites, followed by fast growth of nuclei. The observed differences in the curves for different concentrations indicate that the nucleation and growth mechanism is mixed/intermediate between these two extreme cases, where for lower AgNO_3_ concentrations (0.1–10 mM) before and after reaching a quasi-constant current value, the system is more similar to the instantaneous nucleation mode, while nucleation changes to progressive as the Ag precursor concentration increases to 10 mM. The discrepancies in the ascending and further parts of the curve indicate additional effects that the simple nucleation and growth model does not describe.^24,54^

**Figure 3:**
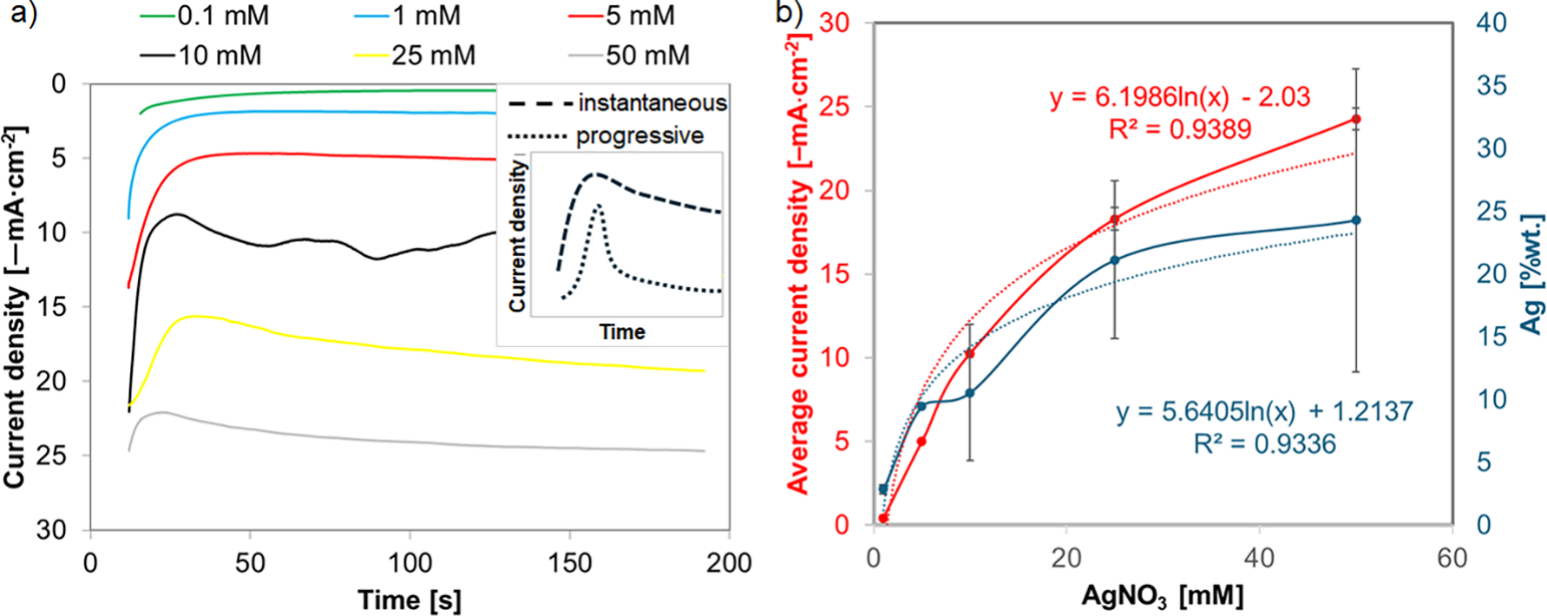
(a) Current density–time curve acquired for TNTs in different concentrations of AgNO_3_ for 180 s at −1.2 V (inset: theoretical current density–time transients predicted by the Scharifker–Hills models for the cases of instantaneous (dashed) and progressive (dotted) nucleation and growth). (b) Graph of the dependence of silver content and recorded current on AgNO_3_ concentration during chronoamperometric silver deposition (dashed—data lines, dotted—regression lines).

Figure 3b shows the logarithmic increase in the silver content and average recorded current with increasing precursor concentration during the chronoamperometric deposition of silver. When the precursor concentration increases to 25 mM, much smaller increases in silver content and recorded current are observed, probably due to saturation (occupation of most of the nanotube surface by silver) of TNT, which is confirmed by SEM images but also due to the tendency of silver to agglomerate, growth of already existing silver particles, and not creation of new nucleation sites (Figure 2). As a consequence, the least stably bound fragments of the three-dimensional Ag structure to the substrate may be washed away.^46^

### The Influence of Silver Deposition Time on the Morphology of the Nanocomposite Produced by Chronoamperometry

SEM images (Figure 4) show the surface morphology of TNT nanocomposite samples with the addition of silver deposited by the chronoamperometric method at a constant precursor concentration, i.e., 1 mM AgNO_3_, a voltage of −1.2 V, and a variable deposition time in the range of 30 to 1200 s. The deposited silver in the form of nanoparticles is located mainly on the surface of the nanotubes, on their edges. At a short electrodeposition time (≤30 s), spherical nanoparticles and flower-like structures were observed (Figure 4a), which then dispersed on the substrate while maintaining the given current conditions, which resulted in a uniform distribution of nanosilver on TNTs in the form of spherical nanoparticles (Figure 4b). As the process continued, the nanoparticles merged into larger clusters, and from some of them, small Ag dendrites with short and numerous branches grew (Figure 4c). Over time, Ag dendrites became larger with multilevel branches. At a longer deposition time (*t* ≥ 900 s), dendrites expanded laterally and vertically, forming a multilayer coating composed of highly branched dendritic fractal nanostructures (micrometer-sized) composed of nanoplates and nanoparticles (Figure 4d–f). Significantly, the thickness of the produced dendrites also increased over time. Spherical microparticles and microhemispheres were also observed (Figure 4e,f). These results agree with the results of Cheng et al.,^44^ who presented the evolution of silver morphology deposited by the chronoamperometric method from 20 to 240 s. The spontaneous self-assembly process of nanosilver is analogous to the mechanism of TiO_2_ nanotube formation on titanium, which at the initial stage resembles a disordered porous structure. The equalization of the current distribution between the pores promotes their automatic ordering, which leads to the obtaining of a regular nanotube structure. However, further anodization causes the chemical dissolution of the nanotube tops, which results in the formation of a disordered layer of thin needle-like structures called ″nanograss″ in their upper parts, which can block some of the nanotube holes—similar to the agglomerated fractal dendritic structures and microparticles that arise as a result of morphological evolution.^7^

**Figure 4:**
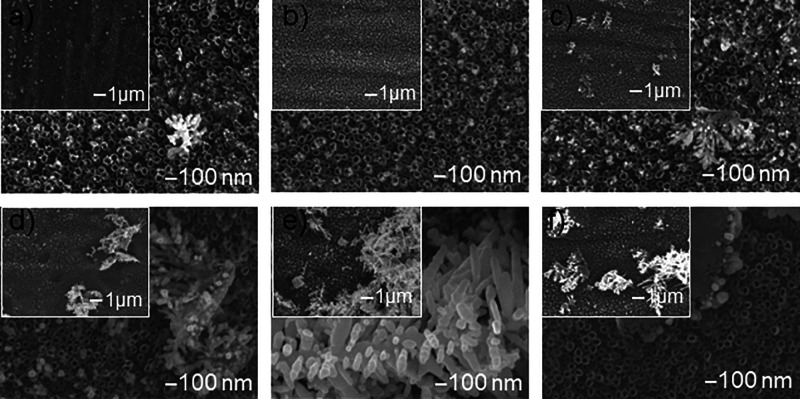
SEM images of the surface morphology of TNT nanocomposite samples with silver additive deposited by the chronoamperometric method at a voltage of −1.2 V in 1 mM AgNO_3_ and variable deposition times: (a) 30, (b) 150, (c) 300, (d) 600, (e) 900, and (f) 1200 s.

[Fig fig5]a shows the current density–time curve acquired for TNTs at different electrodeposition times in 1 mM AgNO_3_ at −1.2 V. The initial rapid decrease in current density corresponds to the immediate process of nucleation of the first nuclei and growth of silver crystallites, followed by stabilization of current density. The recorded oscillations at extended deposition time indicate that the hydrogen evolution reaction occurs together with Ag deposition.^6^ Similar curves were recorded for copper deposition on the stainless steel surface, and the extension of deposition time caused the extension of the plateau current density–time, which occurred after 60 s of deposition,^55^ similar to the present study. Moreover, these studies showed that the size of copper particles increases with the increase in deposition time.

**Figure 5:**
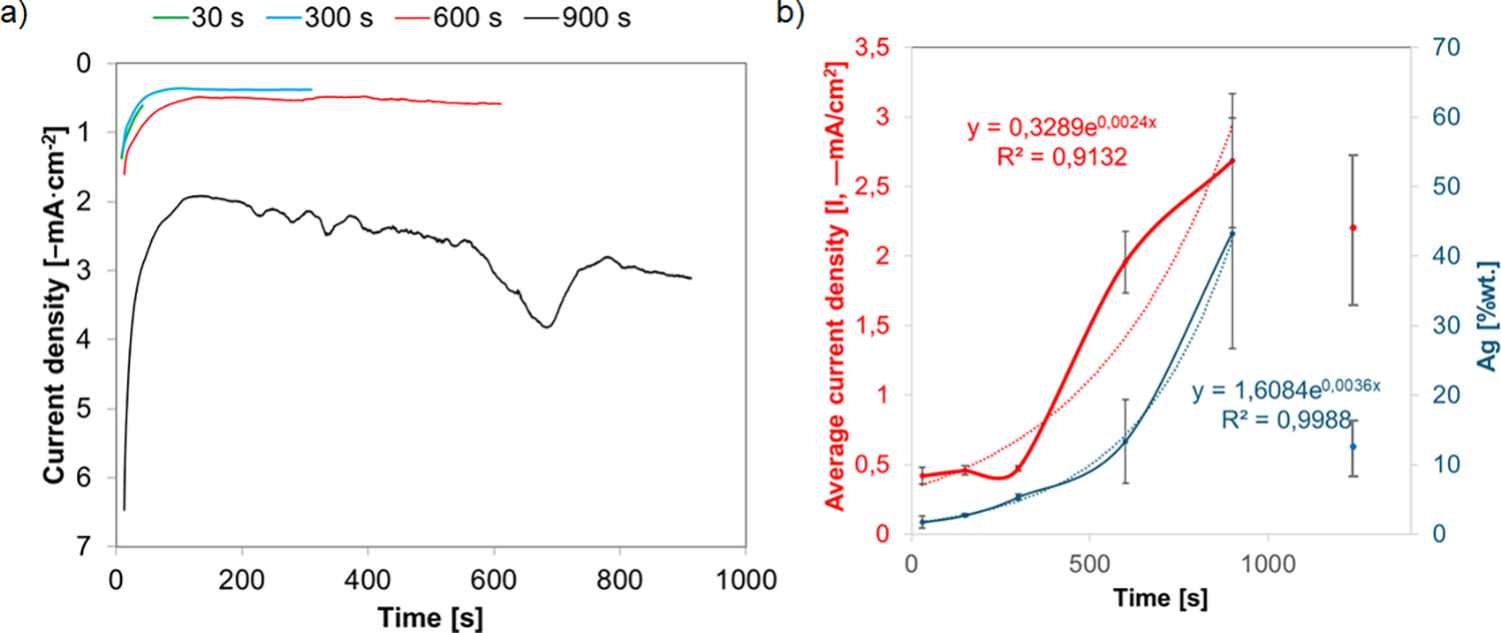
(a) Current density–time curve acquired for TNTs in different times of electrodeposition in 1 mM AgNO_3_ at −1.2 V. (b) Graph of the dependence of silver content and recorded current on time of chronoamperometric silver deposition (dashed—data lines, dotted—regression lines).

Figure 5b shows the exponential increase of silver content and the average value of the recorded current with the increase of the chronoamperometric silver deposition time. When the deposition time exceeds 900 s, the recorded average current decreases, as does the silver content. This is probably due to the tendency of silver to aggregate, which is why the TNT surface is not completely saturated (insets, Figure 4). However, the vertical growth of dendrites is visible, with subsequent branches that are likely to be washed away after the electrodeposition process, which was performed on all samples according to the described methodology.

### The Influence of Voltage on the Morphology of the Nanocomposite Produced by Chronoamperometry

SEM images (Figure 6) show the surface morphology of TNT nanocomposite samples with the addition of silver deposited by the chronoamperometric method at a constant precursor concentration, i.e., 1 mM AgNO_3_ and time 1500 s, and a variable voltage in the range of −0.4 to −1.2 V. Silver deposited in the electrodeposition process at a potential of −0.4 to −0.6 V occurred on the TNT surface mainly in the form of Ag deposit but also in the form of nanoflowers (Figure 6a) and spherical nanoparticles (Figure 6b). As established in our previous studies,^7^ nucleation and subsequent growth of the formed nuclei occur above the EN potential of 0.2 V (called nucleation overpotential). With the increase in nucleation overpotential, more deposited spherical silver nanoparticles and morphological evolution of dendritic structures were observed. As described above, two fundamental processes occur during electrodeposition: nucleation and growth. The working electrode must be more negatively polarized to start electrochemical deposition than the potential at which the reduction starts. The precursor cations are reduced to Ag^0^ adatoms (atoms adsorbed to the surface), which combine to form nuclei as the process progresses. However, if they do not reach the critical size, which is stable, then they dissolve. Therefore, the potential must be more negative than the critical potential to achieve a stable size.^7^ In potentiostatic (and voltammetric) methods, dense and evenly distributed nuclei can be quickly generated at a high nucleation overpotential and low concentration of the nanoparticle precursor,^56^ which is confirmed by Figure 6c–e and Figure 2a,b. Interestingly, Cajiao Checchin et al.^36^ indicate that micropatches can also be formed due to anodic polarization at a potential of +0.8 V. However, as confirmed by SEM photos, cathodic reduction allows Ag structures of various shapes and sizes to be obtained by modifying time, concentration, and applied voltage.

**Figure 6:**
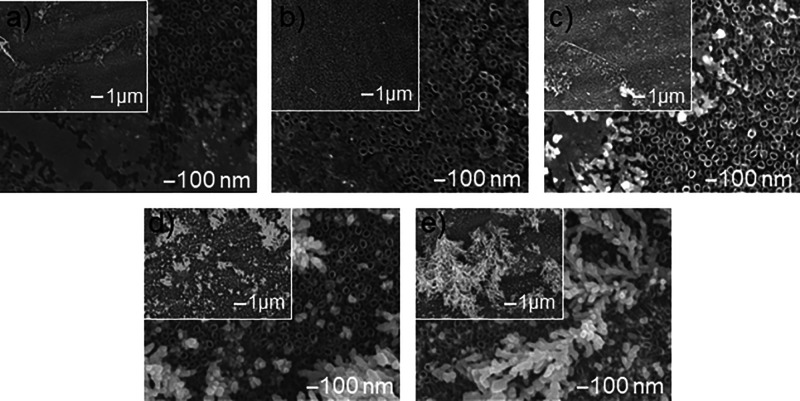
SEM images of the surface morphology of TNT nanocomposite samples with silver additive deposited by the chronoamperometric method in 1 mM AgNO_3_ solution for 1500 s and variable voltage: (a) −0.4, (b) −0.6, (c) −0.8, (d) −1.0, and (e) −1.2 V.

[Fig fig7]a shows the chronoamperometric curves of Ag deposition for 1500 s on a TNT substrate at potentials ranging from −0.4 to −1.2 V. It was found that the recorded current density curves were similar to minor differences probably resulting from the imperfections of the TNT substrate. Initially, the current density gradually decreased, corresponding to forming the first nuclei on the electrode. Then, after 150 s, a relatively stable current was obtained due to further nucleation and growth of Ag on TNTs. Figure 7b shows the changes in the silver content and the average value of the recorded current with the voltage increase applied during the chronoamperometric deposition of silver (with second-order polynomial regression). When the deposition voltage is −1.2 V, the recorded current is comparable to that recorded during deposition at −1 V, similarly to the silver content. These results, as mentioned earlier, are from the saturation of the substrate, i.e., the biased aggregation of silver, which results in easy rinsing of multilevel branches that are less firmly bound to the TNT substrate.

**Figure 7:**
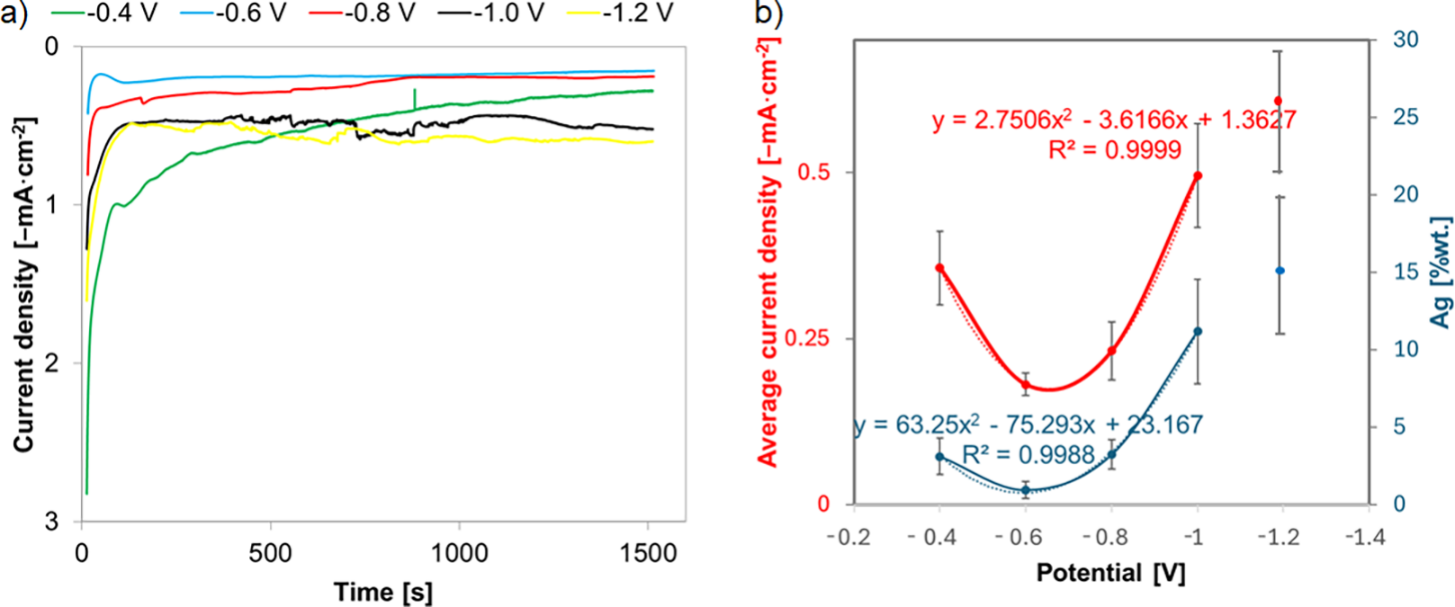
(a) Current density–time curve acquired for TNTs in 1 mM AgNO_3_ for 1500 s and variable voltage: from −0.4 to −1.2 V. (b) Graph of the dependence of silver content and recorded current on the given potential during chronoamperometric silver deposition (dashed—data lines, dotted—regression lines).

Large error bars in the case of the Ag chronoamperometric deposition method refer to the standard deviation of the silver content (EDS analysis) for samples for which a precursor concentration of 5 mM and higher (Figure 3b) or a deposition time of 600 s and longer (Figure 5b) or voltages higher than −0.8 V (Figure 7b) were used, i.e., for TNT structures with agglomerated silver in the form of nanoleaves and micrometric structures. Since the nature of the agglomeration process is the growth of existing silver particles and not the creation of new nucleation sites, it is obvious that the silver concentration on these samples will be uneven, resulting in the height of the error bars. The above differences may also be reflected in the recorded current response, i.e., the observed oscillations of the measured signal.

### The Influence of Precursor Concentration on the Morphology of the Nanocomposite Prepared by Cyclic Voltammetry

Taking into account the literature reports^56–59^ indicating that with the increase of nucleation overpotential, the number of nucleation nuclei increases and the size of deposited nanoparticles decreases, and also that deposition of nanostructures in the range of potentials at which hydrogen adsorption and desorption additionally occurs (on the principle of competition) significantly limits the growth of NPs, but at the same time increases the active surface of composites, a potential in the range of −1.25 to −0.7 V was selected to investigate the evolution of silver morphology using the cyclic voltammetry method. Silver deposited in the electrodeposition process using the cyclic voltammetry method in the form of spherical nanoparticles (Figure 8a,b) is located (similarly to the chronoamperometric method) mainly on the surface of nanotubes, on their edges. In both electrochemical deposition methods, lower precursor concentrations, i.e., in the range of 0.1 to 1 mM, favor the formation of nanometric structures. Densely and uniformly distributed nanoparticles are observed on the titanium nanotubes (TNT), with particle size increasing as the precursor concentration rises. The nanoparticles agglomerate at concentrations exceeding 5 mM into flower- and leaf-like structures. This further growth into dendritic-shaped microparticles leads to the obstruction of TNT channels, resulting in a marked decrease in their specific surface area. Comparing the insets in Figures 8 and 2, it can be seen that the dendrites produced by the CV method form a structure consisting of a larger number of dendrite layers, quasicontinuous (quasicontinuous film) almost on the entire TNT surface. SEM images also confirm that using the CV method, it is impossible to obtain a smooth and tightly closed silver coating on TNTs, as the morphology of TiO_2_ nanotubes determines this inhomogeneity.

**Figure 8:**
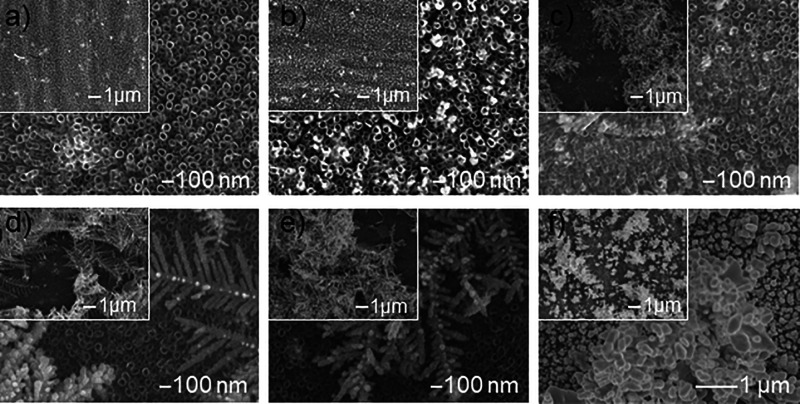
SEM images of the surface morphology of TNT nanocomposite samples with silver additive deposited by cyclic voltammetry at −1.25 to −0.7 V and 25 cycles and varying concentrations of silver ion precursor in the form of AgNO_3_: (a) 0.1 mM, (b) 1 mM, (c) 5 mM, (d) 10 mM, (e) 25 mM, and (f) 50 mM.

[Fig fig9]a shows the last recorded Ag deposition cycles for different precursor concentrations ranging from 0.1 to 50 mM. The inset in Figure 8a shows an example voltammogram recorded for Ag deposition on TNTs from 1 mM AgNO_3_ solution for 25 cycles, which shows that with the increase in the number of cycles, the current densities decrease. Their relative stabilization occurred after about four cycles, while their incomplete overlap indicates the remaining number of nucleation centers after reversing the scanning direction. Figure 9b shows the power increase in the silver content and changes in the recorded current (with second-order polynomial regression) with the increase in precursor concentration. Comparing the %wt. of deposited silver, the CV method (Figure 9b, ranging 1.4 ± 0.6–63.2 ± 8.6%wt.) is more than two times more efficient than the CA method (Figure 3b, ranging 2.2 ± 0.4–24.3 ± 9.9%wt.), which is most likely due to the fact that subsequent CV cycles (inset Figure 9a) do not overlap completely due to the remaining nucleation centers.^7^ The decrease in the average current intensity with the duration of the process can be explained by the nucleation and growth of Ag deposits.^60^ Increasing the precursor concentration from 0.1 to 50 mM increases the recorded current by one order of magnitude.

**Figure 9:**
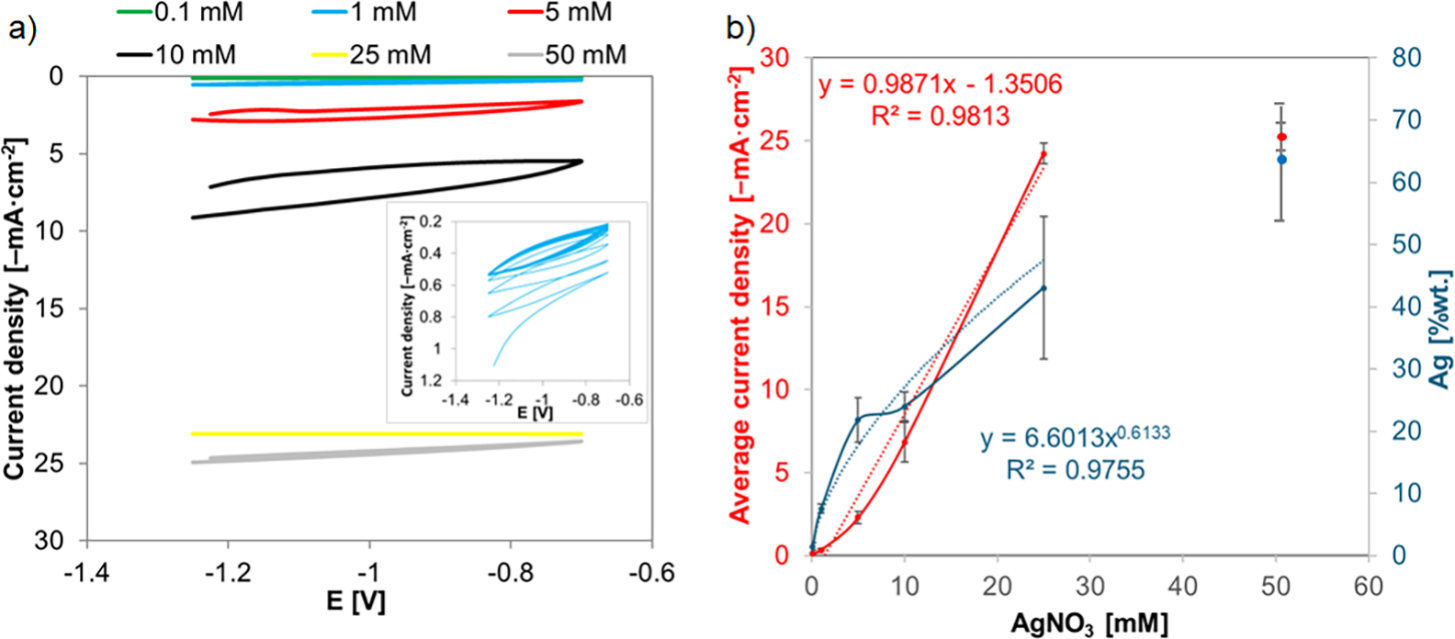
(a) The last of the recorded Ag deposition cycles for different precursor concentrations (inset: voltammogram from silver deposition in 1 mM AgNO_3_ for 25 cycles). (b) Graph of the dependence of silver content and recorded current on AgNO_3_ concentration during voltammetric silver deposition (dashed—data lines, dotted—regression lines).

### The Influence of the Number of Cycles on the Morphology of the Nanocomposite Produced by Cyclic Voltammetry

In the evolution of the morphology of silver deposited on TNTs by cyclic voltammetry in 1 mM AgNO_3_ solution in the range of 5 to 75 cycles, with a constant voltage range from −1.25 to −0.7 V (Figure 10), after five deposition cycles, the presence of uniformly distributed nanoparticles on the TNT surface and single agglomerates in the shape of leaves/flowers is observed. The nanoparticles are uniformly distributed after approximately 20 deposition cycles, while dendritic structures appear after 35 deposition cycles, which due to their growth occupy an increasingly larger surface area of the TNTs. After 75 deposition cycles, the presence of microhemispheres is observed.

**Figure 10:**
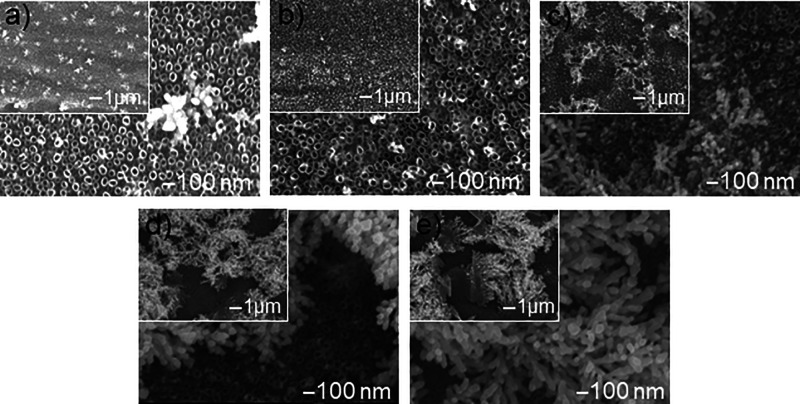
SEM images of the surface morphology of silver-doped TNT nanocomposite samples deposited by cyclic voltammetry in 1 mM AgNO_3_ solution at −1.25 to −0.7 V and a variable number of cycles: (a) 5, (b) 20, (c) 35, (d) 50, and (e) 75.

[Fig fig11]a shows the last Ag deposition cycles on TNTs for a series of samples with variable cycle numbers. Figure 11b shows the exponential increase of the silver content and the recorded current with an increasing number of cycles during the voltammetric deposition of silver. When the number of cycles exceeds 50, the average value of the recorded current and the silver content (considering the standard deviation) does not change, resulting from silver’s tendency to aggregate. Therefore, the vertical growth of dendrites probably destabilizes the structure, and the branches that are the most distant from TNTs may be washed away.

**Figure 11:**
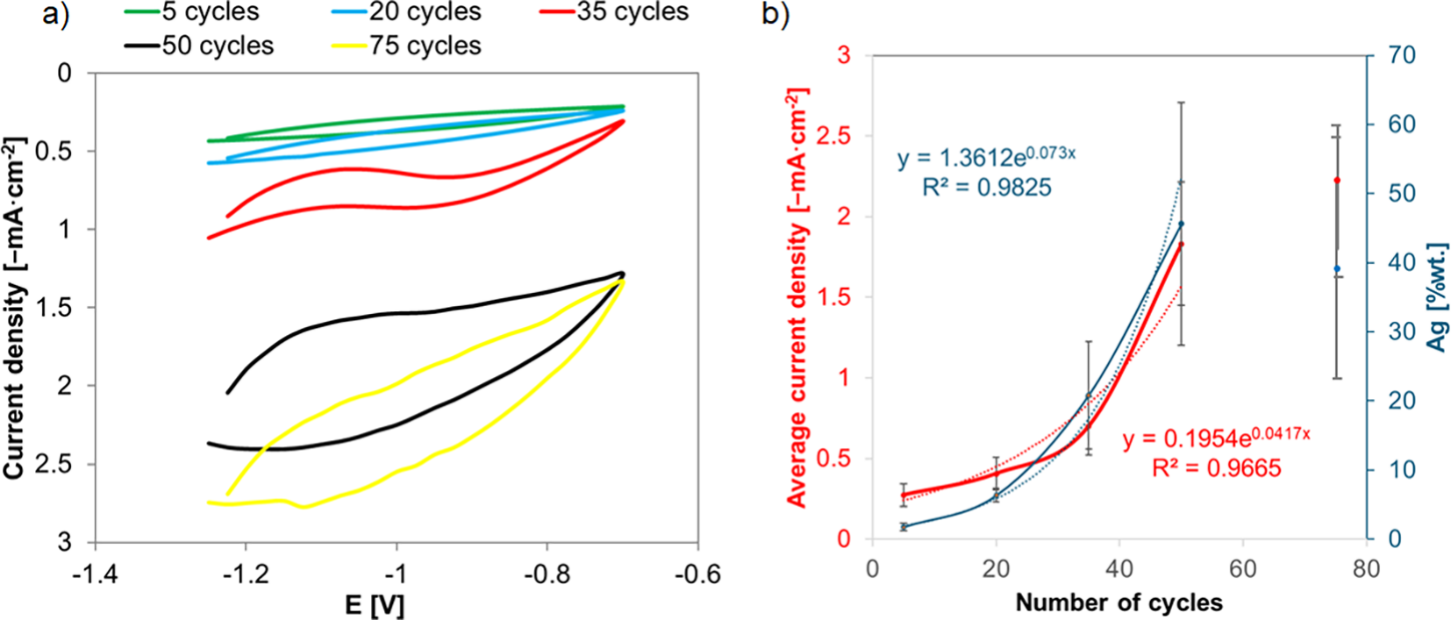
(a) The last of the recorded Ag deposition cycles for deposition in the range of 5 to 75 cycles. (b) Graph of the dependence of silver content and recorded current on the number of cycles during voltammetric silver deposition (dashed—data lines, dotted—regression lines).

Large error bars in the case of the voltammetric method are observed analogously to the chronoamperometric method for samples for which the precursor concentration of 5 mM and higher (Figure 9b) or for the number of cycles higher than 20 (Figure 11b) was used, i.e., for TNT structures with agglomerated silver in the form of nanoleaves and then micrometric structures, while the smallest error bars are noted for samples for which silver was deposited in the form of near-spherical nanostructures relatively evenly covering the TNT surface.

From the above, it follows that for the applied electrochemical methods of silver deposition, one universal mechanism of evolution of the morphology of the deposited silver can be proposed according to the scheme presented in Figure 12. Single particles are formed from the initial nanometric clusters and then evolve into short dendrites, nanoflowers, and nanoleaves. As the process continues, the dendrites grow into multilevel branched fractal structures resembling fern leaves, eventually evolving into micrometric particles resembling semicircles. The initial stage is the nucleation phase, initiated when the first silver ions are reduced to metallic Ag nuclei, which grow into larger nanoclusters through Ostwald ripening. These, in turn, grow through the effects of aggregation and coalescence, creating structures resembling trees—dendrites. Many authors have found that the growth of silver dendrites is anisotropic. In the crystal growth process, the resulting structures’ final morphology depends on the formation conditions departing from thermodynamic equilibrium. Rapid nucleation and growth of nanocrystals, which are nonequilibrium processes, contribute to forming more complex structures with hierarchical morphology. The diffusion-limited aggregation model can be used to interpret the nonequilibrium fractal growth process.^45,61,62^

**Figure 12:**
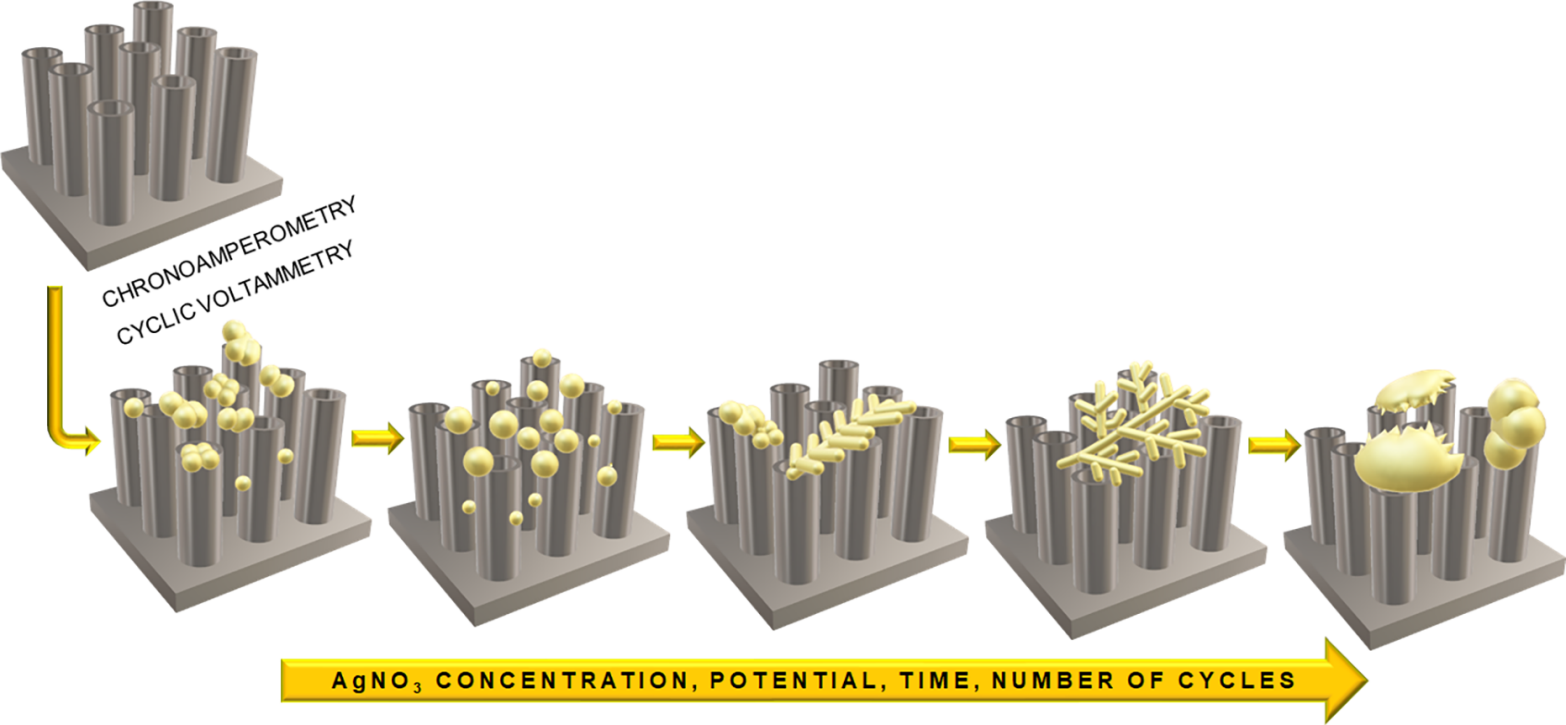
Scheme of evolution of the morphology of silver deposited by electrochemical methods on TiO_2_ nanotubes.

## Conclusions

This paper describes the complex aspects of metal electrodeposition on titanium dioxide nanotube solid electrode surfaces through studies of the metal precursor solution concentration, process duration, and current conditions. The deposition of silver on TNT platforms by chronoamperometric electrodeposition was carried out at five different voltages (−0.4, −0.6, −0.8, −1.0, and −1.2 V), six different AgNO_3_ concentrations (0.1, 1, 5, 10, 25, and 50 mM), and six different deposition times (30, 150, 300, 600, 900, and 1200 s), while the voltammetric method was carried out at the potential ranging from −1.25 to −0.7 V and six different AgNO_3_ concentrations (0.1, 1, 5, 10, 25, and 50 mM) and with the number of cycles from 5 to 75 (5, 20, 35, 50, and 75). It has been proven that both single and hierarchical, fern-leaf-like clusters of silver can be easily, rapidly, and controllably synthesized on TNTs by using electrochemical methods. Based on SEM analyses, it was demonstrated that a universal mechanism exists for the evolution of silver morphology, regardless of the applied electrodeposition method. With the increase of the precursor concentration, deposition time, and applied voltage, silver grows and reorients according to the following scheme: initially, the deposited Ag has the form of clusters of Ag nanoparticles resembling nanoflowers and nanoleaves in their shape; as the process continues, after the current is distributed evenly on the surface (which is the result of the nature of the electrochemical process and the morphology of nanotubes), spontaneous ordering occurs, and the structure is uniformly/homogeneously covered with silver nanoparticles, which over time, through aggregation, create short nanorods/dendrites, and then create multilevel branches. Finally, Ag evolves into microsized quasi-spherical and microsemicircular particles that are connected to each other. The presented morphological changes occurring during electrodeposition correlate with the recorded changes in current density: generally speaking, low current densities (up to several mA·cm^–2^) predispose to the formation of spherical and quasi-spherical nanometric structures or nanoflowers/leaves, while higher densities result in the formation of hierarchical structures that grow to micrometric dimensions. Considering the weight percentage of deposited silver, the voltammetric method is more efficient than the chronoamperometric method, which is likely due to the fact that subsequent CV deposition and stripping cycles do not completely overlap, leaving remaining nucleation centers, resulting in a higher overall deposition rate.

## Supplementary Material



## Notes

Summary of silver electrodeposition conditions on TiO_2_ depending on the expected Ag particle morphology (PDF)

## Abbreviations

AgNO_3_: silver nitrate
AgNPs: silver nanoparticles
CA: chronoamperometry
CV: cyclic voltammetry
TNT: TiO_2_ nanotubes
SEM: scanning electron microscope
