# Supplementary material for: From Nano- to Microsilver: Morphology Control and Shape Evolution of Facile One-Step Electrochemical Synthesis of Silver Particles on TiO2 Nanotubes
Source: Langmuir. 2025 Jun 20;41(25):15963–73. doi: 10.1021/acs.langmuir.5c01022 (PMC12224319; doi:10.1021/acs.langmuir.5c01022)
Supplement: Supplementary file 1 [file la5c01022_si_001.pdf]

Supporting Information

From Nano to Micro Silver: Morphology Control and Shape Evolution of Facile One-step Electrochemical Synthesis of Silver Particles on TiO_2 Nanotubes

Marta Nycz[1], Katarzyna Arkusz[1]*

[1]Department of Biomedical Engineering, Faculty of Engineering and Technical Sciences, University of Zielona Gora, Prof. Z. Szafrana 4, 65-516, Zielona Gora, Poland

*** Corresponding Author:** m.nycz@iimb.uz.zgora.pl, ORCID: 0000-0001-7383-141X

Contents of the Supporting Information:

Number of pages: 3

Number of figures: 0

Number of schemes: 0

Number of tables: 1

Table of contents:

Tab. S1. Tabulated summary of silver electrodeposition conditions on TiO_2 depending on the expected Ag particle morphology……………………..…………..…………………………..S2

Table S1 indicates the parameters of voltammetric and chronoamperometric silver production on TiO_2 nanotubes, resulting in the formation of an Ag/TNTs structure with a specific morphology. Part of the data was taken from the authors' earlier publication, which focused on the comparison of the effects of producing spherical silver nanoparticles on TiO_2 nanotubes using electroreduction and sputter deposition methods, as well as the electrochemical characteristics of AgNPs/TNTs composites, as indicated by the * expanded in the title of Table S1.

Table S1. Tabulated summary of silver electrodeposition conditions on TiO_2 depending on the expected Ag particle morphology. *based on results from[1].

Expected structure	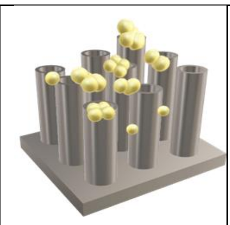	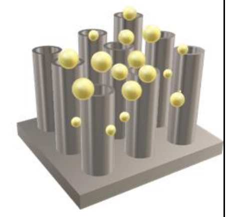	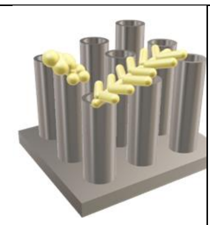	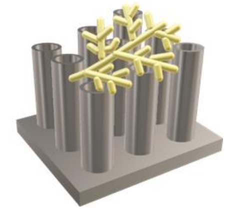	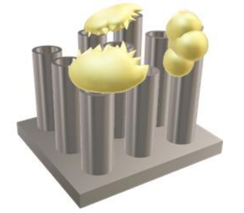
Cyclic voltammetry	0.1 mM $AgNO_3$<br>25 cycles<br>or<br>1 mM $AgNO_3$<br>5 – 15* cycles	1 mM $AgNO_3$<br>20 – 25 cycles	5 mM $AgNO_3$<br>25 cycles<br>or<br>1 mM $AgNO_3$<br>35 cycles	10 – 25 mM $AgNO_3$<br>25 cycles<br>or<br>1 mM $AgNO_3$<br>50 cycles	min. 50 mM $AgNO_3$<br>Min. 25 cycles<br>or<br>1 mM $AgNO_3$<br>min. 75 cycles
Chronoamperometry	1 mM $AgNO_3$<br>30 s<br>–1.2 V<br>or<br>0.1 mM $AgNO_3$<br>180 s<br>–1.2 V<br>or<br>1 mM $AgNO_3$<br>1500 s<br>–0.4 V	1 mM $AgNO_3$<br>60* – 180 s<br>–1,2 V<br>or<br>1 mM $AgNO_3$<br>1500 s<br>–0,6 V	1 mM $AgNO_3$<br>300 s<br>–1,2 V<br>or<br>5 mM $AgNO_3$<br>180 s<br>–1,2 V<br>or<br>1 mM $AgNO_3$<br>1500 s<br>–0,8 V	1 mM $AgNO_3$<br>600 – 900 s<br>–1,2 V<br>or<br>10 – 25 mM $AgNO_3$<br>180 s<br>–1,2 V<br>or<br>1 mM $AgNO_3$<br>1500 s<br>–1,0 – –1,2 V	1 mM $AgNO_3$<br>1200 s<br>–1,2 V<br>or<br>50 mM $AgNO_3$<br>180 s<br>–1,2 V

REFERENCES

1. Nycz, M.; Arkusz, K.; Pijanowska, D. G. Influence of the Silver Nanoparticles (AgNPs) Formation Conditions onto Titanium Dioxide (TiO_2) Nanotubes-Based Electrodes on their Impedimetric Response. *Nanomaterials* **2019**, *9*, 1–16.
